# Competition over Personal Resources Favors Contribution to Shared Resources in Human Groups

**DOI:** 10.1371/journal.pone.0058826

**Published:** 2013-03-08

**Authors:** Jessica L. Barker, Pat Barclay, H. Kern Reeve

**Affiliations:** 1 Department of Neurobiology and Behavior, Cornell University, Ithaca, New York, United States of America; 2 Department of Psychology, University of Guelph, Guelph, Ontario, Canada; Hungarian Academy of Sciences, Hungary

## Abstract

Members of social groups face a trade-off between investing selfish effort for themselves and investing cooperative effort to produce a shared group resource. Many group resources are shared equitably: they may be intrinsically non-excludable public goods, such as vigilance against predators, or so large that there is little cost to sharing, such as cooperatively hunted big game. However, group members' personal resources, such as food hunted individually, may be monopolizable. In such cases, an individual may benefit by investing effort in taking others' personal resources, and in defending one's own resources against others. We use a game theoretic “tug-of-war” model to predict that when such competition over personal resources is possible, players will contribute more towards a group resource, and also obtain higher payoffs from doing so. We test and find support for these predictions in two laboratory economic games with humans, comparing people's investment decisions in games with and without the options to compete over personal resources or invest in a group resource. Our results help explain why people cooperatively contribute to group resources, suggest how a tragedy of the commons may be avoided, and highlight unifying features in the evolution of cooperation and competition in human and non-human societies.

## Introduction

Conflict over the division of resources is widespread across taxa, from intraorganismal conflict in algae [1] to competition among animal social group members [2]–[4], including humans [5], [6]. For individuals in social groups, two types of resources can potentially be divided: personal resources that each individual has produced for itself, such as food from a solitary hunt; and group resources consisting of individuals' pooled contributions, such as large prey caught in a cooperative hunt, which are shared, often equally, among all group members [7], [8]. In this study, we investigate how the potential to take other people's personal resources may increase the benefit of cooperatively producing group resources that are equitably divided, a topic of considerable social and political importance in human groups [9].

Some resources to which group members contribute energy or time (hereafter, group resources) are non-excludable and non-rivalrous (public goods, *sensu strictu*): that is, they are intrinsically accessible to all group members, are non-depletable, and thus there is no competition over their division. For example, in an insect society, workers' investments in raising the queen's offspring benefits all group members according to their relatedness to the queen [10]; likewise, the benefits of predator inspection or vigilance, e.g. in guppies *Poecilia reticulata* [11], are automatically shared. Similarly, many group resources shared by humans are intrinsically non-contestable public goods, such as clean air, public radio, and defense against other groups [12].

Other group resources are depletable or potentially monopolizable, for example food shared among chimpanzees *Pan troglodytes* [13] and humans [14]. However, in practice many of these resources are shared equitably, such that they are functionally non-contestable. Such equal sharing of depletable resources may occur for several reasons. The group resource may be so large, e.g. big game from a cooperative hunt or plentiful fish stocks, that it is too costly for one individual to monopolize (i.e., not economically defendable [12], [15]). Allowing others to obtain shares of a group resource may also be low cost if an individual becomes satiated (i.e., tolerated theft [16]–[18]). Alternatively, group members may benefit by investing in mechanisms to prevent competition over shared resources, such as policing in both human and non-human societies [19]–[23], and social institutions in human groups [24], [25]. Indeed, human groups are noteworthy for the degree to which potentially monopolizable group resources are shared equally [26], [27].

In contrast, individuals may invest energy in producing personal resources for themselves, which are often excludable, depletable “private goods”. In human groups, these resources include wealth and food [12]: for example, the !Kung own small prey from individual hunts [28], and the Machiguenga rarely cooperate with group members to obtain food [29]. In these cases, an individual may be able to increase the size of her own personal resources by selfishly taking personal resources from others (even from kin [30]). For example, in non-human primates, harassment over food is common, e.g. in macaques *Macaca fascicularis* [31], squirrel monkeys *Saimiri boliviensis* [32] and chimpanzees *Pan troglodytes* [33]. Members of some human societies similarly attempt to steal others' personal resources, e.g. in the Dobu [34] and Mikea [35] societies. In response, individuals in both human and non-human groups benefit by investing in resource defense [36] in order to reduce their group-mates' selfish efforts. For example, hymenopteran workers eat eggs selfishly laid by other workers in order to lay their own [37]; humans also invest in policing to protect their resources.

Investing in taking others' and defending one's own resources (hereafter, “competition”) is costly to all group members, constituting an “arms race” of investment in manipulation and counter-manipulation that reduces the amount that individuals invest in their personal or group resources [19]. There is therefore a trade-off between investing in group resources, personal resources and competition: thus the division of contestable personal resources may affect individuals' investments in group resources. We hypothesize that competition over individuals' personal resources increases the benefit of contributing to a non-contested group resource of which all group members obtain an equitable share: this is because an individual will reliably gain a benefit from greater investment in the group resource, but any benefit from greater investment in personal resources may be reduced by others' investments in competition. Under this hypothesis, we predict that: (1) given that individuals have the opportunity to contribute to a group resource, contributions will be higher when competition over personal resources is possible versus when it is prevented; and (2) given that there is competition over personal resources, individuals' payoffs will be higher when they have the opportunity to contribute to a group resource.

We investigated this hypothesis both mathematically and empirically. Firstly, we used a game theoretic “tug-of-war” model [38] to predict individuals' optimal investments in cooperation and competition. Secondly, we tested the verbal and mathematical predictions in human groups, using laboratory economic games based on a “public goods” game [39]–[43]. Such games are often used as a model for the collective action problem of resource-sharing, and can measure the structural and/or psychological factors promoting cooperation [29], [39]–[43]. We use them here to empirically examine the effects of these different payoff structures in promoting cooperation. Please note that (1) it is always an empirical question whether people respond to payoff structures as the models predict they will [44], and (2) we are agnostic about which specific psychological mechanisms (“proximate causes”) are being triggered [45]–[47].

In a basic tug-of-war model, an individual obtains a fraction of resource proportional to its investment in competition relative to other group members. Individuals' investments in competition diminish the absolute amount of the contested resource; i.e., there is a trade-off between increasing one's own fraction and reducing the total amount of resource. Tugs-of-war have been used to analyze the division of group resources in both invertebrates, particularly social insects [48], and vertebrates, e.g. mountain gorillas *Gorilla beringei beringei* [49], cooperatively breeding cichlids *Neolamprologus pulcher* [50] and humans [51], [52]. Previous models have investigated the effect of competition over shared resources, and have shown how policing to enforce contribution to and equal division of a shared resource affects investment in tug-of-war competition [20], [23], [53], [54]. In contrast, here we apply the tug-of-war framework to the division of personal resources in order to investigate how the presence of competition affects the costs and benefits of contributing to an equally shared resource.

## Model

### Assumptions

Each player in the game starts with an amount of available effort which she can choose to expend in producing resources. We assume that maximization of resources corresponds to fitness maximization, where effort invested in resource acquisition represents a fitness cost, and the resources obtained confer a fitness benefit. Players can invest effort in producing shared group resources, which we assume are divided equally among group members (see [51] for effects of unequal division), or in personal resources, which they keep for themselves. In the game with competition, players can also invest effort attempting to take others' personal resources, and in defending their own personal resources from being taken. In this scenario, we assume that group members do not differ in their ability to invest in taking or defense. We also assume that investments in taking and defense are equally effective (see text S1 for the case where this assumption is relaxed). Finally, in both of the games we present here, players are non-relatives, but we show in text S1 that including kinship does not qualitatively change the results of the model.

### No competition over personal resources

We start by considering a classic public goods game, which is the basis of many laboratory studies on cooperation [39]–[43] and into which we will incorporate tug-of-war competition. In this basic model, each individual has a total available effort of value *v*, of which she cooperatively invests an amount *y* in a producing a shared group resource, and selfishly invests *v*-*y* into producing a private resource kept for herself. Investing effort in the group resource produces collective benefit: all contributions to producing the group resource are summed and multiplied by *k*, and the resulting resources are split equally among the *n* members of the group.

We seek the optimal contribution effort y* that maximizes the amount of resources an individual obtains; i.e., maximizes its fitness. To do this, we consider the amount of resources *w_y_* obtained by a focal individual contributing *y* in a population of *n*−1 other individuals contributing y*:
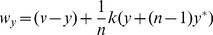
(1)


At the Nash equilibrium, the focal individual's contribution *y* is equal to all others' contributions y*, and d*w_y_*/d*y*  = 0; this allows us to solve for y*. We find that y* is an endpoint maximum: that is, individuals should contribute either all (*v*) or none (0) of their effort towards producing collective resources, but should not contribute an intermediate amount. (For details on finding the endpoint maxima, see text S1.) We determine that when *k*>*n*, y* = *v* (individuals maximize their fitness by contributing all of their effort towards producing group resources, and obtain a payoff *w_y_* = *kv*), but when *k*<*n*, y* = 0 (individuals maximize their fitness by investing all of their effort in producing personal resources, and obtain a payoff *w_y_* = *v*). This basic finding replicates previous theoretical work on public goods contributions, e.g. [39], [40], and we build on it below.

### “Tug-of-war” over personal resources

We now consider the case where any personal resource in which an individual has invested may be taken by other group members. An individual invests effort *x* in attempting to defend her personal resources from others, and *z* in trying to take others' personal resources; i.e., a “tug-of-war” competition [38] over resources produced for oneself. Each investment *z* is spread evenly among the other group members; that is, an effort *z* corresponds to an effort 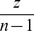 in taking from any given player. The effectiveness of a given effort in resource defense relative to effort in taking from others is given by the factor *b*.

We seek the optimal values x*, y* and z*, and again do so by considering a mutant individual adopting the strategies *x*, *y* and *z*, in a population of *n*−1 others adopting the optimal strategies x*, y* and z*. The fraction of personal resources that the focal individual defends, *d*, is determined by her investment in resource defense relative to the other players' investments in taking from her:
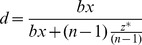
(2)


The fraction of personal resources that the focal individual takes from another player, *t*, corresponds to her investment in taking from that player relative to that player's resource defense and *n*−2 other players' efforts in taking from him:
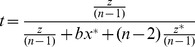
(3)


After her investments above, the focal player has an amount *v*-*x*-*y*-*z* of effort to invest in producing her personal resource. The total amount of resources that this focal individual obtains, *w_xyz_*, is therefore a fraction *d* of what she produced for herself plus a fraction *t* of what each other player produced for himself, plus an equal share of the contributions to the group resource (we assume equitable division of the group resource because this is frequently the case in human societies [7], [14], [55]; see [51] for the effects of non-equitable division):
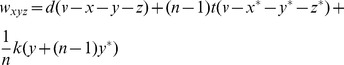
(4)


We then find the partial derivatives δ*w_xyz_*/δ*x*, δ*w_xyz_*/δ*y* and δ*w_xyz_*/δ*z*; we evaluate these derivatives at *x* = x*, *y* = y* and *z* = z*, when all individuals are adopting the optimal strategies, and set them equal to zero. As above, we find an endpoint maximum for y*: when *k*>1, y* = *v* (individuals should contribute everything to producing the group resource, and obtain a payoff *w_xyz_* = *kv*), and when *k*<1, y* = 0 (individuals should contribute nothing). When *k* = 1, an individual's fitness is not affected by the amount she contributes. Thus, the condition favoring contributions to the group resource in this game with a tug-of-war over personal resources (*k*>1) is much more permissive than that in the standard public goods game with no tug-of-war (*k*>*n*).

The case where *k*>1 applies to any situation in which the pooled efforts that group members contribute yield a synergistic benefit of cooperation. For example, if individuals must work together to accomplish something they cannot achieve individually, then *k* is effectively greater than 1. This assumption would apply to cases such as when (a) group hunters bring down large prey that they cannot bring down alone, (b) group members must move massive objects against friction or gravity to build shelters, and (c) group members must match the size of rival groups to protect resources from the latter.

For cases where y* = 0 (including when there is simply no group resource to which players can contribute), we simultaneously solve δ*w_xyz_*/δ*x* = 0 and δ*w_xyz_*/δ*z* = 0 for the values x* and z*. We verify that these correspond to fitness maxima by checking that the second derivatives are negative. When investments in defense and taking are equally effective (*b* = 1, as in our experiment; see text S1 for solutions when *b*≠1), we find that the optimal strategies are:
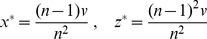
(5)


Thus, a player should invest *n*−1 times as much effort in taking (z*) than in defense (x*), since each investment in taking is spread among the *n*−1 other players. Substituting these values for x* and z* into *w_xyz_*, we find that at equilibrium an individual obtains a payoff *w_xyz_* = 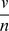. Because this payoff is smaller than *kv*, players end up doing much better by investing in the group resource whenever one is available.

### Summary of the model's predictions

The model predicts that contributing effort to producing a group resource will be evolutionarily stable under a wider range of conditions when there is competition over personal resources than when there is no such competition over personal resources (*k*>1 versus *k*>*n*, respectively). The result is a potentially large inflation in payoffs for all because conflict has been avoided: contributing to a group resource provides an escape from the costly arms race represented by a mutual tug-of-war. This result holds whether or not kinship is included in the model (see text S1).

## Methods

### Ethics statement

All methods were approved by the Cornell University Institutional Review Board for Human Participants (ID# 0907000684). We recruited participants (118 females and 66 males of various ethnicities; mean age: 20.85 years ±s.e 0.30 years) from the Cornell University community using posters and mailing lists. We obtained informed written consent from participants before they began the experiment, and participants completed instructions and a test of understanding before starting each experimental condition in the game (see text S2).

### Overview of economic game

Participants played the experimental economic game in groups of four people. Each group of participants played two experimental conditions (see below), programmed using z-Tree software [56]. Each participant played the game at a computer terminal visually isolated from the other players, and all decisions were confidential.

Each condition contained 10 rounds; players received 100 “lab dollars” (L$) each round, which they could invest in different ways depending on the condition. This approach of choosing monetary investments represents an individual deciding how to invest time and energy into different activities (see model above). Participants did not know how many rounds of the game they would play. At the end of the game, lab dollars were exchanged for cash payoffs in US dollars (L$300:US$1, plus a baseline payment of US$2), with a mean payoff of US$9.10± s.e. US$0.18, with exact earnings depending on participants' decisions during the game.

### Public goods game condition: Contribute and Keep (CK)

Each participant could divide her money among two options: contribution to the group (C) and setting aside personal money to keep for herself (K). These options represent individuals investing effort in producing group resources and private resources respectively. (In the experiment, these were called the “group fund” and “production fund”, to avoid framing problems). As in a standard public goods game, contributions to the group fund were doubled and redistributed equally among all players [39], [40], [42], [43], [57], [58]. Each player's payoff at the end of each round was therefore equal to the money she kept plus her quarter share of the doubled group fund (equation 1). Information about all players' contributions and payoffs was displayed on each participant's computer screen before she moved onto the next round.

### Tug-of-war condition: Keep, Take and Defend (KTD)

In this condition, a group fund was not available for players to contribute to. Each participant could keep money for herself (K), but could also invest in attempting to take the personal money that others kept (T) and defending her own personal money from others' attempts to take it (D). These investments constitute a “tug-of-war” over players' personal resources, corresponding to strategies *z* and *x* respectively in the model (see above); in the experiment they were called “extraction” and “retention”. Each player's payoff at the end of each round was equal to the personal money she defended plus the money she took from others (i.e., equation 4 with *y* = y* = 0). As in the Contribute and Keep condition (CK), participants saw a computer screen with all players' investments in each fund and their payoffs before they started a new round of the game.

Of the money kept for herself, the amount that a participant retained at the end of the round depended on her investment in defense relative to other players' investments in taking from her (equation 2). For example, if player A invested L$30 in defense and the other three players invested a total of L$50 in taking from her, then A would end up with a 3/8 share [30/(30+50)] of her own personal money.

A player's investment in taking from others was divided among the three other group members, as in the model above: for example, a L$30 investment meant a player invested L$10 in taking from each other player's personal money. The amount of money that a participant received from another player's personal keepings depended on her own investment in taking from him relative to his investment in defense, and the other players' investments in taking (equation 3). For example, if player B invested L$30 in defense, player A invested L$10 in taking from him, and the other two players invested L$20 each in taking from him, player A would get a 1/8 share (10/[10+30+20+20]) of B's personal money. If no-one invested in taking from anyone else, each person would keep all of her personal money.

### Public goods game plus tug-of-war condition: Contribute, Keep, Take and Defend (CKTD)

Participants playing this condition had four options. Each player could invest in keeping money (K) for herself; taking (T) from others' kept amounts; and defending (D) her own kept amount from others' taking, as in the tug-of-war game. Additionally, each player could invest in contributing (C) to a group fund that was doubled and divided equally, as in the public goods game. That is, investments in taking and defending apply only to the money players kept for themselves, and not to the money contributed to the group fund. Each participant's payoff at the end of each round was thus equal to one quarter of the doubled group fund, plus personal money she defended, plus others' personal money that she took (equation 4).

### Comparisons of experimental conditions and statistical analyses

Each group played two experimental conditions, with the order of conditions counterbalanced between groups. This allowed us to make the following comparisons.

#### Comparison 1: CKTD versus CK (i.e., public goods game with/without tug-of-war)

26 groups played experimental conditions CK and CKTD; that is, the possibility to invest in the tug-of-war differed between conditions.

#### Comparison 2: CKTD versus KTD (i.e., tug-of-war with/without group resource)

20 groups played experimental conditions KTD and CKTD; that is, the possibility to contribute to the shared group fund differed between conditions.

We use a within-subject design in our empirical test. The model predicts that, since *n* = 4 and *k* = 2 in our game, (1) in the CKTD condition, participants should contribute everything (y* = *v*); (2) in the CK condition, participants should invest in keeping everything (y* = 0); and (3) in the KTD condition, participants should adopt the stable intermediate values of x* and z* (effort in defense and taking respectively; equation 5). However, people typically avoid extreme decisions in laboratory economic games [59], even when doing so is not optimal, and thus are unlikely to invest the absolute values predicted by any model [39], [60]. Therefore, the relevant predictions here are the relative differences in people's decisions between experimental conditions [51], [59].

For our statistical analysis, we treated each group of 4 participants as an *n* of 1, to control for interdependence within groups. We analyzed the data using a general linear model (SPSS 17.0) with experimental condition and round as within-groups variables, and with the order of conditions as a between-groups variable. For two groups in the CKTD versus CK experiment, minor problems arose with the instructions program during the game (quiz questions appearing at the wrong time or not at all), but excluding these groups from the analysis did not affect the results.

## Results

### Comparison 1: CKTD versus CK (with/without tug-of-war)

Contributions to the group fund were significantly higher when people could invest in the tug-of-war (CKTD condition: L$53.4 ± s.e. L$4.3) than in the condition without a tug-of-war (CK condition: L$39.0± s.e. L$4.6; *F*(1,24)  = 10.86, *p* = 0.003). This is all the more striking given that participants had the opportunity to spread their money among four options in the CKTD condition, compared with two in the CK condition: having more options would normally dilute participants' investments among those options, and yet participants still invested more in contributing the group fund, and less in keeping money for themselves, when the tug-of-war was present (see text S3 for further evidence of this).

**Figure 1:**
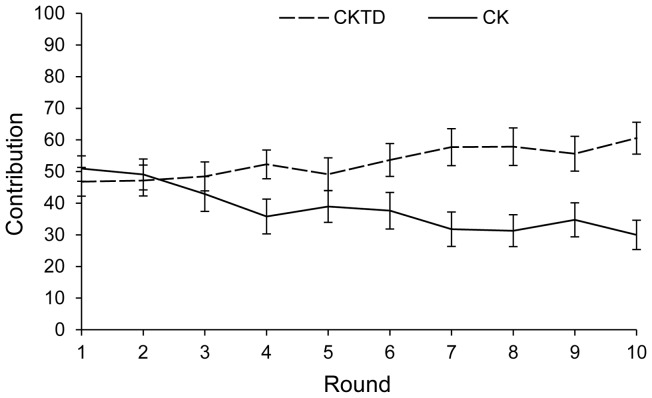
Contributions in games with and without tug-of-war competition. Mean (± s.e.) contributions in lab dollars (L$) to the group resource in each round when there is a public goods game plus tug-of-war (CKTD condition, dashed line) versus a public goods game only (CK condition, solid line).

There was a significant interaction between experimental condition and round number (*F*(9,216)  = 10.20, *p*<0.001): in the CK condition, contributions fell over time (*F*(9,216)  = 10.12, *p*<0.001), whereas contributions increased in the CKTD condition (*F*(9,216)  = 3.04, p = 0.002). The possibility of investing in a tug-of-war thus means that contributions do not fall (figure 1). Order was counterbalanced across sections; see text S3 for an analysis of order effects on contributions over time.

**Figure 2:**
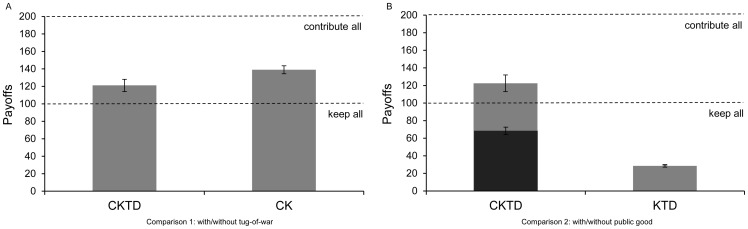
Participants' payoffs in all experimental conditions. Mean (± s.e.) payoffs in lab dollars (L$) per round (means are calculated over 10 rounds of each condition). If all players had contributed all of their money to the group resource each round, each player's payoff would be L$200; if all players had kept all of their money for themselves each round, each player's payoff would be L$100. (a) Comparison of games with and without the options for tug-of-war competition (CKTD and CK respectively). (b) Comparison of games with and without the option to contribute to a group resource (CKTD and KTD respectively). Black bar shows players' adjusted payoffs if money contributed to the group resource had not been doubled (i.e., if *k* = 1).

Despite the higher contributions in the CKTD condition, participants earned significantly higher payoffs in the CK condition (L$139.0 ± L$4.6) compared to the CKTD condition (L$121.1 ± L$7.0; *F*(1,24)  = 8.85, *p* = 0.007, no effect of order; figure 2a); that is, people were worse off when they had the option to invest in a tug-of-war, because the tug-of-war used up resources.

In sum, this comparison of the CKTD versus CK conditions shows that when people had the option to invest in competition over personal resources, (1) they contributed more to the group resource, (2) these contributions increased over time, and (3) they earned less money than when competition was not an option.

### Comparison 2: CKTD versus KTD (with/without group resource)

Participants' payoffs were significantly higher when they could contribute to a shared group resource (CKTD condition: L$123.6 ± s.e. L$9.6) than in the experimental condition without the option for contribution (KTD condition: L$28.6 ± s.e. L$1.4; *F*(1,18)  = 90.77, *p*<0.001; figure 2b). There was no effect of order or interaction with order on any of the results regarding participants' payoffs (all *F*s <1).

People's higher payoffs in the CKTD condition may simply be because money contributed to the group fund, unlike that kept in a personal fund, was not subject to the tug-of-war, or because it was doubled before being equally divided among participants (*k* = 2). In order to distinguish between these alternatives, we can hypothetically adjust people's payoffs to determine what they would have obtained if the group resource had not been doubled (*k* = 1). Participants' adjusted payoffs in the CKTD condition (L$68.5 ± s.e. L$4.2) are still significantly higher than in the KTD condition (*F*(1,18)  = 79.23, *p*<0.001; figure 2b), suggesting that the difference in payoffs is not simply due to the group resource being doubled. Please note, however, that participants made their decisions in light of the fact that the group resource was doubled, and thus this latter analysis does not explain participants' behavior during the game, but rather provides a potential reason why their payoffs were higher in the CKTD condition.

In the KTD condition, people kept significantly more money, and invested significantly more in the tug-of-war, than in the CKTD condition (all *F*s >24, all *p*s <0.001, table 1; no effect of order or interaction with order: all *F*s <1). This is not surprising, as people had only three investment options in the KTD condition, compared with four in the CKTD condition. Instead, the relevant values are the relative investments: how much people kept for themselves and invested in the tug-of-war, out of the total not contributed to the group fund (i.e., the amount invested in each of keeping, taking and defense, divided by the sum of these three investments). There was no significant difference between people's relative investments in the two conditions (all *F*s <2; all *p*s >0.2). In the KTD condition, people invested more in taking than in defense or amounts kept (*F*(1,17)  = 5.21, *p* = 0.017; table 1), as predicted by the model. Taking money is unlikely to be a form of moralistic punishment: people who took more from their group-mates tended to contribute a lower proportion of their remaining money to the group fund (see text S4).

**Table 1:** Investments in the different options in games with and without a group resource.

Investment	CKTD condition: absolute amount a	CKTD condition: relative amount b	KTD condition c
Kept for self	13.4±2.2	31.3±2.1	28.6±1.4
Investment in taking	16.9±2.0	38.9±2.1	41.3±2.5
Investment in defense	14.7±2.5	29.8±2.4	30.1±1.6
Contribution	55.1±5.5	n/a	n/a
Total d	100	100	100

a Participants invested significantly lower absolute amounts in keeping, taking and defense in CKTD than in KTD (all *F*s >24; all *p*s <0.001).

b There was no significant difference in the relative amounts invested in each of the three options in CKTD versus KTD (all *F*s <2; all *p*s >0.2).

c Participants invested significantly more in taking money than in either defense or keeping money (*F*(1,17) = 5.21, *p* = 0.017) in the KTD condition. In the CKTD condition, the results were in the same direction but were not significant (relative amounts: *F*(1,17)  = 3.15, *p* = 0.069; absolute amounts: *F*(1,17)  = 2.60, *p* = 0.103).

d Totals may not sum exactly to 100 due to rounding.

Mean (± s.e.) investments in lab dollars in the experimental conditions with and without the option to contribute to a public good (CKTD and KTD respectively). Participants could spread their money among four options in the CKTD condition, compared to three in the KTD condition; in order to control for this, the “relative amount” column shows the amounts participants kept and invested in taking and defense relative to the sum of investments in these three options.

In sum, this comparison of the CKTD versus KTD conditions shows the following. (1) When people had the opportunity to invest in contributing to an equally shared group resource, they obtained higher payoffs than when this was not an option. (2) This increase in payoffs is unlikely to be merely due to the doubling of the contributions to the group resource. (3) The option to contribute did not affect how people distributed their endowment among the other investment options.

## Discussion

### Contributions to the group resource

People invested more in contributing to public good production when they could also invest in tug-of-war competition (CKTD condition), as compared to when there was no tug-of-war (CK condition); this was true despite having more investment options in the former experimental condition. In addition, the presence of a tug-of-war prevented the decline in contributions over time; such a decline is otherwise typical in public goods games when such competition is absent (CK condition) [39]–[41], unless there is an opportunity for reputation or punishment [57], [58], [61]. This finding can plausibly be explained by people choosing to contribute more when their own resources were at risk of being taken, since competition over the group resource was not permitted.

Thus, this empirical result supports the game theoretic prediction that in a game with a tug-of-war (CKTD condition), players should contribute to the group resource above a lower threshold return on their investment than in a game without a tug-of-war (CK condition). Our evolutionary model shows when such behavior would be adaptive, and our experimental results show that people do indeed respond to such incentives and use the provision of public goods as a type of protected resource. We are agnostic about whether such behavior represents evolved preferences, rational thinking, reinforcement-based learning, or other such proximate psychological mechanisms.

Two potential criticisms of this result are unlikely to be problematic. Firstly, people may have contributed more in response to being confused by having more options; however, they were tested on their understanding of the game before they were allowed to begin the experiment. Secondly, people contributed in all experimental conditions in the laboratory game even when this was not the optimal strategy predicted by the model for the parameters of our experiment. This is very common in experimental games [39]–[41] and may occur because people avoid extreme strategies in laboratory games, regardless of whether they are optimal [59]. Our within-subjects empirical design controls for this by allowing us to analyze the relative differences between experimental conditions.

### Tugs-of-war and the tragedy of the commons

Unlike in games with punishment, in this experiment a participant could not target her investments in resource defense and taking towards a specific other player. In addition, people who took more money from others tended to contribute a smaller proportion of their remaining endowment to the group (see analysis in text S4). This suggests that, in contrast to other cases where people spent money in order to reduce the payoffs of the highest earners [62], [63] or lowest contributors [57], [64], investments in taking and defense here were not moralistic sanctions but were simply made in order to maximize one's own personal resources relative to others. This leads to a costly arms race, where people benefit by escalating their competitive investments. Investing in competition reduces the amount of money one can keep or contribute, thus resulting in a tragedy of the commons where everyone is worse off than if no-one had invested in competition [65]–[67]. The results of the laboratory game reflect this, with people receiving lower payoffs in the CKTD condition than the CK condition.

The lowest payoffs overall were in the KTD condition, where people did not have the option to contribute to a group resource; that is, payoffs in KTD < CKTD < CK. A potential explanation for the higher payoffs in CKTD versus KTD is that all contributions to the group resource were doubled, whereas money in players' personal resources was not. However, had contributions not been doubled (*k* = 1; “adjusted earnings”), people would still have earned more in the CKTD condition. Players' higher payoffs are thus better explained by the non-contestability of the group resource: equal division precluded people spending money in a competitive arms race. This suggests that the opportunity to contribute to a group resource over which there is no competition (as is the case for many shared resources: [7], [14], [48]) may provide a solution to the tragedy of the commons caused by investing effort in a tug-of-war over personal resources.

### Competition and the evolution of shared group resources

In many scenarios outside the laboratory, humans invest effort in acquiring personal resources that can potentially be taken by others, as when people in the Hadza [68] and Mikea [35] societies attempt to hide private shares of food. People also frequently invest effort in producing common resources that are shared among all group members; if these resources are not intrinsically non-excludable, competition over them is nonetheless frequently precluded. For example, there may be no net cost to sharing if the resource is large enough, as in a Lamalera whale hunt [29] or Meriam turtle hunt [14]; alternatively, the social cost of not sharing may be high if the resource is divided in public, as among the Nayaka [69]. Such group resources act as “banks” where individuals' investments are protected from scramble competition (i.e., converted into public goods) and possibly even gain value (*k*>1); indeed, this is likely why monetary banks were initially established [70], [71]. The collective benefits from such group resources may simply be an incidental byproduct of individuals following their self-interest in producing resources from which they are guaranteed at least some share, and are not necessarily driven by prosocial preferences for others [72].

The “tug-of-war arms race over personal resources” hypothesis described here provides one explanation for contribution to producing non-contestable group resources: that is, the net cost of investing in defending one's own personal resources and attempting to take others' outweighs the cost of contributing to resources that are shared by all, analogous to models of food-sharing by harassment in non-human primates [32], [73], [74]. This hypothesis is not mutually exclusive with other explanations such as reciprocity and risk reduction in uncertain environments [75]. In the present experiment, we isolated the effects of competition by having participants: (1) make anonymous decisions, thus reducing the potential for reciprocity and reputation; (2) receive a fixed amount of money each round, thus not have to buffer uncertainty; and (3) never become satiated, and thus suffer a cost from contributing to others. Please note that this tug-of-war hypothesis provides an ultimate explanation [45], [46] for contribution to shared group resources – that is, based on resource acquisition in different payoff structures – and does not attempt to elucidate individuals' proximate psychological motivations.

In sum, the implications of this study are threefold. Firstly, the theoretical and empirical results suggest that the opportunity to contribute effort to producing an equally shared group resource, especially one that has the potential to earn interest, helps to limit mutually destructive competition over personal resources. Secondly, this finding is not specific to humans, but applies to any social groups in which individuals can invest effort in competing over personal resources [38] and contributing to shared group resources. For example, costly competition among hymenopteran workers over male production [76], [77] may select for contribution to the queen's reproductive success as a non-excludable shared resource. By drawing on approaches developed in different disciplines, such as tug-of-war theory and economic games, we can uncover universal evolutionary principles governing the balance between cooperation and conflict across the animal kingdom. Finally, much environmental and social conflict in human societies arises over the contribution to and division of personal and shared resources [78], [79] and elucidating the evolutionary explanations for these behaviors can help us more effectively manage them [80]–[83].

## Supporting Information

Text S1 **Supplementary game theoretic analyses for the model.** (a) Finding endpoint maxima. (b) Including relatedness among players (*r*>0). (c) Effectiveness of taking relative to defending resources (*b*≠1).
(DOCX)

Text S2 **Documents for participants in the economic game.** (a) Consent form. (b) Instructions. (c) Test of understanding.
(DOCX)

Text S3 **Supplementary statistical analyses for CKTD versus CK experiments.** (a) Controlling for the number of investment options. (b) Keeping money. (c) Effects of order on contributions.
(DOCX)

Text S4 **Supplementary statistical analyses for CKTD versus KTD experiments: taking money and moralistic punishment.**
(DOCX)
