# Supplementary material for: Competition over Personal Resources Favors Contribution to Shared Resources in Human Groups
Source: PLoS One. 2013 Mar 8;8(3):e58826. doi: 10.1371/journal.pone.0058826 (PMC3592809; doi:10.1371/journal.pone.0058826)
Supplement: Text S1 — Supplementary game theoretic analyses for the model. (a) Finding endpoint maxima. (b) Including relatedness among players (r>0). (c) Effectiveness of taking relative to defending resources (b≠1). (DOCX) [file pone.0058826.s001.docx]

**Supporting text S1: supplementary game theoretic analyses**

**a. Finding endpoint maxima**

We found no intermediate solution to d*w_y_*/d*y* = 0, which means there is no value of *y* between 0 and *v* that maximizes a player’s payoff (i.e. there is no solution 0<*y**<*v*). Thus, a player’s optimal strategy is to contribute either all (*y**=*v*) or none (*y**=0) of her resources; that is, *y** is an endpoint maximum. We determine which of these boundary solutions (*y**=0 or *v*) maximizes a player’s payoff by examining d*w_y_*/d*y*: if d*w_y_*/d*y*<0, then the lower boundary gives the fitness maximum; if d*w_y_*/d*y*>0, the upper boundary does. We find that d*w_y_*/d*y* = 0 when *k*/*n*=1, such that when *k*>*n*, players should contribute everything (*y**=*v*), and when *k*<*n*, they should contribute nothing (*y**=0).

We apply the same procedure to *w_xyz_* (i.e. the payoff in game with a tug-of-war) to determine which boundary value maximizes a player’s payoff.

**b. Including positive relatedness among players**

If players are related to each other by a coefficient of relatedness *r*, then the inclusive fitness, rather than the personal fitness as given by equations 1 and 4 in the main text, should be maximized. The inclusive fitness of the focal mutant is just its own personal fitness plus the personal fitnesses of each of the *n*-1 non-mutants multiplied by *r*.

In the game with no competition over personal resources, we again find an endpoint maximum for *y**. Players should invest all their effort in contribution (*y**=*v*) when *k*(*r*(*n*-1)+1)>*n*, and below this threshold should invest all their effort in personal resources (*y**=0). This threshold for contribution is more permissive when *r* increases: that is, contribution is favored under a wider range of values when players are more highly related. (Note that the threshold for contribution reduces to *k*>*n* when *r*=0, as in the main text.)

For the game with competition, the local inclusive fitness maxima for *x* and *z* are given by:

Equations S1

$$x*=\frac{(1-r)(n-1)v}{n^{2}}$$

$$z*=\frac{(1-r)(n-1)^{2}v}{n^{2}}$$

That is, they are simply the previous solutions multiplied by 1-*r*: thus, as *r* increases, players should invest less in the costly tug-of-war. Again, we find an endpoint maximum for *y**: when *k*>1, *y**=*v* (individuals should contribute everything, and obtain a payoff *w_xyz_*=*kv*), and when *k*<1, *y**=0 (individuals should contribute nothing); this condition holds regardless of the value of *r*. Thus, adding a positive relatedness among group members to the tug-of-war does not qualitatively change the prediction that the presence of a tug-of-war increases the incentive for contribution to a group resource.

**c. Effectiveness of defending relative to taking resources**

The effectiveness of a given investment in resource defense (*x**) relative to an investment in taking from others (*z**) is given by the factor *b*. We simultaneously solve δ*w_xyz_*/δ*x*=0 and δ*w_xyz_*/δ*z*=0 for the fitness-maximizing values *x** and *z** (see main text), and find that:

Equations S2

$$x*=\frac{(b(n-1)-n+2)(n-1)v}{b^{2}(n-1)^{2}+b(n^{2}-1)-n(n-2)}$$

$$z*=\frac{b(n-1)^{2}v}{b^{2}(n-1)^{2}+b(n^{2}-1)-n(n-2)}$$

When *b*=1, these values simplify to the values for *x** and *z** given in the main text (equations 5). In addition, note that the numerator of *x** is only greater than zero when $b>\frac{n-2}{n-1}$. Therefore, if$b\leq\frac{n-2}{n-1}$, the optimal strategy is to invest *x**=0 in defense, because defending one’s resources is ineffective. If *b* is above this critical value, the optimal investment in defending (*x**) relative to taking (*z**) is:

Equation S3

$$\frac{x*}{z*}=\frac{b(n-1)-n+2}{b(n-1)}$$

When *b*=1, this simplifies to $\frac{1}{n-1}$: that is, a player should invest *n*-1 times more in taking from others than in defending. This is because any investment in taking (*z**) is spread among the *n*-1 other group members (see main text).

In order to determine how the relative investments in defending and taking vary with *b*, we evaluate the derivative of equation S3 with respect to *b*:

Equation S4

$$\frac{d\frac{x*}{z*}}{db}=\frac{n-2}{b^{2}(n-1)}$$

Given that *n*>2, this derivative is always positive, and thus as *b* increases (that is, resource defense increases in effectiveness relative to taking), a player should increase her investment in defense relative to taking.
