# Supplementary material for: Competition over Personal Resources Favors Contribution to Shared Resources in Human Groups
Source: PLoS One. 2013 Mar 8;8(3):e58826. doi: 10.1371/journal.pone.0058826 (PMC3592809; doi:10.1371/journal.pone.0058826)
Supplement: Text S2 — Documents for participants in the economic game. (a) Consent form. (b) Instructions. (c) Test of understanding. (DOCX) [file pone.0058826.s002.docx]

**Supporting text S2: documents for participants**

a. Consent form

b. Instructions

c. Test of understanding

**Group Decision-Making Consent Form**

You are invited to participate in a research study of the decisions that people make in social situations. Your participation in this study is voluntary. We ask that you read this form and ask any questions you may have before agreeing to be in the study.

**Background Information**: The purpose of this study is to examine the sorts of decisions that people make within groups. Many researchers look at the sorts of decisions that people make with money or other currencies that matter to people.

**Procedures:** If you agree to be in this study, we will ask you to interact with other participants in a decision-making task. You will be asked to make a number of decisions involving money, such as whether to keep money or to contribute it towards a group benefit to be shared equally. You will be asked to go through the instructions for the group task, to make decisions in the group task, and then to complete some questions about your experiences in the task. You will also be asked to complete some basic demographic information and some personality questions. The entire experiment will be done in one session and is estimated to take approximately 50 minutes.

**Risks and Benefits of Being in the Study:**

While performing the group task, you may feel annoyed or pleased at other players’ decisions in the task, depending on the particular decisions that others make. Other players may also feel the same about your decisions, but everyone’s decisions are anonymous, so no one will know what decisions you decided to make. You may also feel uncomfortable about some of the questions, but your answers are anonymous also. These risks are unlikely to be greater than the risks that you would encounter in regular social situations. You will be identified to the experimenter and other participants only by a code name, so no one will be able to link you to your decisions or to your questionnaire responses.

You will receive no direct benefits for participating, but your participation will benefit the scientific community because it will add to everyone’s understanding of human decision making and group behavior.

**Compensation:** You will earn money for your decisions. The exact amount that you earn will depend on your decisions and the decisions of the other participants, and will be between $10 and $20.

**Voluntary Nature of Participation:** Your decision whether or not to participate will not affect your current or future relations with the University of Arizona. You may skip questions on the questionnaires and demographic questions if any of them make you feel uncomfortable. If you decide to participate, you are free to withdraw at any time without affecting those relationships. You will only receive money for your decisions if you complete the experiment.

**Confidentiality:** The information from this study will be kept private. When you make your decisions, we will not record any personally identifying information, so no one will be able to link you with your decisions. Data will be kept on a computer in a locked office, indefinitely.

**Contacts and Questions:** The researcher conducting this study is Jessie Barker. Please ask any questions you have now. If you have questions later, you may contact me at jlbarker@email.arizona.edu; 607-254-4370; or W 409 BioSciences West, Department of Ecology and Evolutionary Biology, University of Arizona. If you have any questions or concerns regarding your rights as a subject in this study, you may contact the Institutional Review Board for Human Participants (IRB) at 607-255-5138, www.irb.cornell.edu, or [irbhp@cornell.edu](mailto:irbhp@cornell.edu). You may also report your concerns or complaints anonymously through Ethicspoint by visiting [www.ethicspoint.com](../www.ethicspoint.com%20) or calling toll free at 1-866-293-3077. Ethicspoint is an independent organization that serves as a liaison between the University and the person bringing the complaint, so that anonymity can be ensured.

You will be given a copy of this form to keep for your records.

**Statement of Consent:** I have read the above information, and have received answers to any questions I asked. I consent to participate in the study.

Participant’s Signature ________________________________ Date ______________________

Experimenter’s Signature _________________________ (do not sign – for experimenter to sign)

This consent form will be kept by the researcher for at least five years beyond the end of the study and was approved by the IRB on ____________.

**Instructions**

Each round, you will receive $100 (lab dollars), which you can divide among four places: a Production Fund, a Retention Fund, and an Extraction Fund and a Group Fund.

Production Fund: every $1 you invest in the Production Fund is worth $1. In other words, all money invested in the Production Fund will directly increase your earnings.

However, you will lose a fraction of the money in your Production Fund if other group members invest in Extraction. You can prevent this by investing in Retention. Please see below.

Retention Fund: in order to keep others from Extracting your Production Fund, you can invest in Retention. The proportion of your Production Fund that you get to keep depends on your investment in Retention relative to the other players' investments in Extraction.

For example, if you invest $10 in Retention, and the other three players invest a total of $30 in Extracting your Production Fund, you can keep a ¼ share (10/[10+30]) of your Production Fund. If you invest $30 in Retention and the other three players invest a total of $30 in Extraction, you can keep a ½ share (30/[30+30]) of Production Fund.

If none of the other players invests in Extraction, then you will keep all of your Production Fund, regardless of your investment in Retention.

Extraction Fund: in order to increase your payoff, you can invest in Extraction. The effects of your investment in Extraction are divided among the three other group members. For example, if you invest a total of $30 in Extraction, this means you are investing $10 in extracting money from each other player's Production Fund (i.e. Extraction cannot be targeted towards a specific person). The return on this Extraction depends on others' relative investments in Extraction and Retention.

For example, if one player invests $30 in Retention, you invest $10 in Extraction towards that player, and the other two group members invest $20 each in Extraction towards that same player, you will get a 1/8 share (10/[10+30+20+20] of that player's Production Fund to add to your own payoffs.

If no one invests in Extraction, each player keeps all of his/her Production Fund.

Group Fund: Every $1 you invest in the Group Fund will increase the group's earnings by $2. All money invested in the Group Fund will be added up across all four members, and the experimenter will multiply this amount by two. This money will be divided evenly among all players, regardless of how much they invested in the Group Fund. These earnings are not affected by Extraction.

For example, if everyone invests $100 in the Group Fund ($400 total), this gets doubled so the group earns $800, and everyone gets a $200 share. If no one invests in the Group Fund, then nothing gets doubled, and you only earn what you receive from Production, Retention, and Extraction.

Since you have a maximum of $100 each round (for all four funds), anything you invest in one of these four funds will leave you with less to invest in the other two funds.

Thus, although everyone does well if everyone invests in their Production Funds or in the Group Fund, any single person can try to make more money for him/herself by investing in Extraction and Retention.

**Quiz**

**Question 1**

1a) If everyone invests $100 in their Production Fund, how much does everyone earn?

1b) If everyone invests $100 in Extraction, how much do they all have left to invest in their Production Fund, Retention Fund or Group Fund?

1c) In the case where everyone invests $100 in Extraction, how much does everyone earn?

1d) If everyone invests $100 in Retention, how much does everyone earn?

1e) If everyone invests $100 in the Group Fund, how much does everyone earn?

**Question 2**

2a) If you invest $10 in Retention and the others invest $30 EACH in Extracting from you ($100 total including you), what percent of your Production Fund do you keep?

2b) In the above example (2a), if you had invested $50 in your Production Fund, what would your share of that $50 be?

2c) In the above example (2a), what percent of your Production Fund would each other person get? (Remember that they each invested $30 in Extraction from you.)

2d) In dollars, how much would they each get from your Production Fund?

**True/false**

Investing in your Production Fund provides a payoff that doesn’t depend upon the actions of others (i.e. it provides a fixed-rate payoff)

Investing in the Group Fund provides a payoff that doesn’t depend on the actions of others (i.e. it provides a fixed-rate payoff)

Investing in Retention directly increases your earnings

The more you invest in Extraction, the more you receive from others’ Production Funds

It always pays to invest in Extraction

Investing in Extraction helps you to keep some of your Production Fund
