# Supplementary material for: Competition over Personal Resources Favors Contribution to Shared Resources in Human Groups
Source: PLoS One. 2013 Mar 8;8(3):e58826. doi: 10.1371/journal.pone.0058826 (PMC3592809; doi:10.1371/journal.pone.0058826)
Supplement: Text S3 — Supplementary statistical analyses for CKTD versus CK experiments. (a) Controlling for the number of investment options. (b) Keeping money. (c) Effects of order on contributions. (DOCX) [file pone.0058826.s003.docx]

**Supporting text S3: comparison of CKTD versus CK experimental conditions**

**a. Controlling for the number of investment options**

In order to control for the extra options in the game with a tug-of-war (CKTD), we can analyze people’s contributions relative to the amount they kept for themselves (i.e., amount contributed / [amount contributed + amount kept]), as these were the only options they had in both experimental conditions. In this analysis of relative contributions, people’s relative contributions in the CKTD condition (L$76.1 ± s.e. L$4.0) were significantly higher than the contributions in the CK condition (L$39.0 ± s.e. L$4.6; *F*(1,24)=55.07, *p*<0.001).

**b. Keeping money**

In the standard public goods game (CK condition), the amounts people keep for themselves are simply whatever they do not contribute to the group. Therefore, if people contribute more when there is a tug-of-war (CKTD condition; see main text), this necessarily implies that people keep less for themselves in the CKTD condition ($14.3 ± s.e. L$2.0) than in the CK condition (L$61.0 ± s.e. L$4.6; *F*(1,24)=118.05, *p*<0.001).

Similarly, if we control for the number of investment options in the CKTD condition by analyzing the amount kept relative to the amount contributed (i.e., amount contributed / [amount contributed + amount kept]; see section (a) above), the relative amount people keep is significantly lower in the CKTD condition (L$23.9 ± s.e. L$4.0; *F*(1,24)=55.07, *p*<0.001).

**c. Effects of order on contributions**

The order of experimental conditions (CKTD first versus CK first) had a significant effect on people’s contributions over time (order x round interaction: *F*(9,216)=4.39, *p*<0.001, other effects or interactions involving order are all *F*<1), so we present each order separately below.

When participants played CKTD first, contributions were significantly higher in the CKTD condition (L$54.0 ± s.e. L$5.8) than in the CK condition (L$35.9 ± s.e. L$6.6; *F*(1,12)=7.66, *p*=0.017). There was a significant interaction between experimental condition and round number (*F*(9,108)=3.72, *p*<0.001): in the CK condition, contributions fell over time (*F*(9,108)=6.69, *p*<0.001), whereas contributions did not change in the CKTD condition (*F*<1, n.s.).

When participants played CK first, contributions were not significantly higher in the CKTD condition (L$52.8 ± s.e. L$6.5) than in the CK condition (L$42.1 ± s.e. L$6.6; *F*(1,12)=3.40, *p*=0.090). There was a significant interaction between experimental condition and round number (*F*(9,108)=7.53, *p*<0.001): in the CK condition, contributions fell over time (*F*(9,108)=3.81, *p*<0.001), whereas contributions increased in the CKTD condition (*F*(9,108)=5.10, *p*<0.001).

Thus, despite this interaction, the major results (higher contributions in the CKTD condition, contributions falling over time in the CK condition but not the CKTD condition) are independently replicated in the two different orders. Order was counterbalanced across sessions, with 13 groups playing CKTD first and 13 playing CK first.
