# Supplementary material for: Competition over Personal Resources Favors Contribution to Shared Resources in Human Groups
Source: PLoS One. 2013 Mar 8;8(3):e58826. doi: 10.1371/journal.pone.0058826 (PMC3592809; doi:10.1371/journal.pone.0058826)
Supplement: Text S4 — Supplementary statistical analyses for CKTD versus KTD experiments: taking money and moralistic punishment. (DOCX) [file pone.0058826.s004.docx]

**Supporting text S4: taking money and moralistic punishment**

One possible explanation for taking money is that it is a means of moralistic punishment towards low contributors. This is unlikely to be true for two reasons. Firstly, participants in our experiment could not target their taking towards specific people. Secondly, people who took more money from others tended to be non-contributors themselves. We tested the latter by calculating a “contribution ratio” for each participant based on how much they contributed in the CKTD condition relative to how much they kept, i.e. [C/(C+K)]. This automatically controls for the fact that people who take more will naturally have less remaining to contribute. We carried out a repeated-measures general linear model to see whether these contribution ratios differed between the person in each group who took the most from others, took the second-most from others, the third-most, and the least.

If taking is a form of moralistic punishment, then people who take more should have higher contribution ratios, because such moralistic punishment is usually carried out by cooperators [1,2]. We did not find this in our experiment: indeed, the opposite was true. Players who took the most, second-most, third-most, and least in each group had average contribution ratios of 0.64 (± s.e. 0.05), 0.68 (± s.e. 0.04), 0.76 (± s.e. 0.04), and 0.80 (± s.e. 0.04), respectively; the omnibus F-test was significant (*F*(3,126)=6.62, *p*<0.001), as was the linear contrast (*F*(1,42)=15.16, *p*<0.001). Thus, those who took more than their group-mates also contributed less than their group-mates. Results are very similar if we rank players based on how much they spent on taking plus defending, instead of just taking. The order of conditions had no effect or significant interactions in either case (all *F*s<1).

The results are a little more complicated if we analyze the two experimental comparisons separately (CKTD versus CK, and CKTD versus KTD), given that this factor does interact with how much a person takes relative to other group members (*F*(3,126)=7.16, *p*<0.001). The following results are unlikely to be theoretically interesting, but we present them here for completeness. When participants played the CKTD and KTD conditions, taking was negatively related to the contribution ratio, as it was in the above analysis: players who took the most, second-most, third-most, and least in each group had average contribution ratios of 0.51 (± s.e. 0.07), 0.72 (± s.e. 0.06), 0.74 (± s.e. 0.06), and 0.83 (± s.e. 0.04), respectively; the omnibus F-test was significant (*F*(3,54)=12.79, *p*<0.001), as was the linear contrast (*F*(1,18)=32.34, *p*<0.001). However, when participants played the CKTD and CK conditions, there were different contribution ratios between players who took the most (0.78 ± s.e. 0.06), second-most (0.64 ± s.e. 0.06), third-most (0.79 ± s.e. 0.05), and least within each group (0.78 ± s.e. 0.05), (*F*(3,72)=2.75, *p*=0.049), but these differences were due to quadratic and cubic trends (*F*s(1,24)=4.39 and 4.85, *p*s = 0.047 and 0.038, respectively) instead of a linear increase. Although the latter scenario is more complicated, both cases show that people who take more do not contribute more, so it is unlikely that taking from others is moralistic punishment.

**References**

1. Barclay P (2006) Reputational benefits for altruistic punishment. Evolution and Human Behavior 27: 325–344. doi:10.1016/j.evolhumbehav.2006.01.003.

2. Fehr E, Gächter S (2002) Altruistic punishment in humans. Nature 415: 137–140. doi:10.1038/415137a.
